# Structural implications of traditional agricultural landscapes on the functional diversity of birds near the Korean Demilitarized Zone

**DOI:** 10.1002/ece3.6880

**Published:** 2020-10-12

**Authors:** Jae Hyun Kim, Shinyeong Park, Seung Ho Kim, Keunwon Kang, Bruce Waldman, Myung Hwa Lee, Minhye Yu, Hyunyoung Yang, Hyun Yong Chung, Eun Ju Lee

**Affiliations:** ^1^ School of Biological Sciences Seoul National University Seoul Korea; ^2^ DMZ Ecology Research Institute Paju Korea; ^3^ Department of Integrative Biology Oklahoma State University Stillwater Oklahoma USA

**Keywords:** avian diversity, civilian control zone, conservation planning, drought, dumbeong, irrigation pond, traditional agricultural ecosystem

## Abstract

Bird assemblages are sensitive to changes in landscape composition and the environment, such as those that result from drought. In this study, the relationship between landscape composition and avian functional diversity in traditional agricultural ecosystems in the Civilian Control Zone (CCZ) of Korea was examined. In addition, the resilience of biodiversity to changes in landscape elements resulting from drought conditions was investigated. The traditional agricultural landscape (TAL) of the sites studied was divided into three types: TAL 1 had a high proportion of rice paddies, TAL 2 included large forest areas, and TAL 3 represented areas with drylands. Of these, TAL 1 showed the highest species richness and functional richness, but these measures were most vulnerable to drought. Meanwhile, TAL 2 showed that the bird communities were more tolerant under drought event. This study shows that to conserve and enhance the diversity of birds in traditional agricultural landscapes of Northeast Asia, active management of forest areas is needed to protect bird populations. In addition, commercial pressures to develop this area will require urgent biodiversity conservation plans to protect the unique biodiversity of the Korean CCZ. This study thus provides landscape management guidance for conservation planning.

## Graphical abstract

The species richness (SR) and functional diversity (FD) of birds are affected by traditional landscape practices in the vicinity of the Demilitarized Zone (DMZ). SR and FD are greatest around rice paddies when precipitation patterns are normal, but species assemblages lack resilience to drought. Where forests are abundant, SR and FD little change, even as rice paddies dry in drought conditions. As a result, forests play an important role in the traditional agricultural landscape (TAL), and the maintenance of forests in the TAL is key to maintaining species diversity.

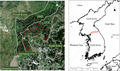

## 1 INTRODUCTION

Biodiversity provides numerous essential services to humans, as has become increasingly apparent in recent decades. Anthropogenic effects on land use represent principal threats to biodiversity conservation (Wilson et al., 2016). Humans depend on agriculture, but agricultural land use dramatically changes surface cover and soil characteristics while some species coexist well with agriculture, in general, intensive agriculture is a principal driver of biodiversity loss (Flynn et al., 2009; Stanton et al., 2018; Tscharntke et al., 2005). Therefore, understanding how land use patterns impact biological diversity and community structure is key to the effective management of agroecosystems (Lee & Martin, 2017).

Rice paddies comprise approximately 11% of agricultural land use worldwide, and over 90% of the world's supply of rice is produced in Asia (FAOSTAT, 2017). Rice paddies can function as alternative natural wetlands and thereby promote biodiversity (Elphick, 2000) with benefits for taxonomic groups such as aquatic plants (Luo et al., 2014), amphibians (Cunha et al., 2015), aquatic insects (Mukai et al., 2005), and birds (Ibáñez et al., 2010). Heterogeneous landscapes, such as those traditionally used in Korean agriculture, can further enhance biodiversity in rice paddies (Kim et al., 2016). Compared with modern intensive agriculture practices, traditional farming maintains greater heterogeneity in landscapes by using small fields, retaining field margins and natural land cover, and planting diverse crops—all practices that have been demonstrated to effectively promote biodiversity (Benton et al., 2003; Martin et al., 2020). Traditional Japanese agricultural landscape, including spatially mixed rice paddies and forests, similarly supports high levels of species diversity (Katoh et al., 2009; Takeuchi, 2003). However, little is known about how traditional Korean agricultural land use, comprising rice paddies and various land cover types, affects wildlife.

Our study area comprises the western part of the Civilian Control Zone (CCZ) near the city of Paju in Gyeonggi‐do Province, South Korea. The DMZ (Demilitarized Zone) and CCZ were designated in 1953 after the Korean War. Although the DMZ remains inaccessible, agriculture has been promoted in the CCZ since the 1970s with the establishment of the Tongilchon village, just 4.5 kilometers south of the Military Demarcation Line (MDL) and within the CCZ. Inhabitants built the village from scratch, cultivating fields to plant beans, ginseng, and rice. Since then, this traditional landscape has been preserved, comprising *dumbeong*, irrigation ponds that supply water to rice paddies, which play an important role in maintaining biodiversity (Kim et al., 2011; Sebastián‐González et al. 2010). Commercial development is highly restricted in the village, fostering the maintenance of traditional agriculture.

Korean *dumbeong* provide shelter for wildlife, even when conditions become extreme (Lee, 2005). As farmland ponds give way to modern irrigation systems, biodiversity can plummet (Lewis‐Phillips et al., 2019). By contrast, *dumbeong,* which irrigate 89% of the rice paddies in the western CCZ (Paju‐si Gunnae‐myeon local office, 2011), effectively protect the natural ecosystem from drought and other environmental stressors. *Dumbeong* serve as a ready reservoir that can supply rice paddies. In this study, we sought to quantify the ecological benefits conferred by *dumbeong*, comparing their effects in normal and drought conditions.

Birds are especially sensitive to water stress, and drought can dramatically change community structure (Smith, 1982). However, current theory is based largely on studies of from terrestrial ecosystems, not wetlands. Even though birds are inextricably subjected to influences of water stress, whether avian communities show higher diversity as alternative wetlands, such as rice paddies, increase in quantity or quality has not been well studied. Korean traditional agricultural landscapes provide a model case in which to examine these questions. Endangered migrating birds, such as the red‐crowned crane and swan goose, overwinter in the CCZ and nearby estuaries (Lee et al., 2007), but species that breed and nest in paddy fields during the summer have gone unstudied. To investigate the bird communities under specific environmental conditions, using functional diversity can be useful as well as traditional taxonomic diversity. Functional diversity is determined by the range of traits in an assemblage and is expected to be responsive to landscape composition or environmental conditions (Hooper et al., 2005; Luck et al., 2012). Therefore, functional diversity may clarify a bird community composition with structural portrayal and may reveal the traits which are effective for surviving in a harsh drought condition. Furthermore, among a bunch of research dealing with functional diversity, few studies have considered the functional indices according to the land use and land cover (LULC) in combination with climate events (but see Weyland et al., 2019). In this study, we examine the resilience of avian community diversity in Korean traditional agricultrual landscape (TAL), when facing up to drought.

We investigated the structure of Korean traditional agricultural landscapeand the response of avian community in accordance with the TAL type under the drought events. We hypothesize that (a) avian taxonomic and functional diversity would respond differently according to the dominant natural elements and anthropogenic elements, (b) the higher proportion of irrigation pond would contribute to the resilience of avian taxonomic and functional diversity against drought events by replenishing water to maintain wetland environment in rice paddies.

## 2 METHODS AND MATERIALS

### 2.1 Bird surveys and TAL units

**Figure 1:**
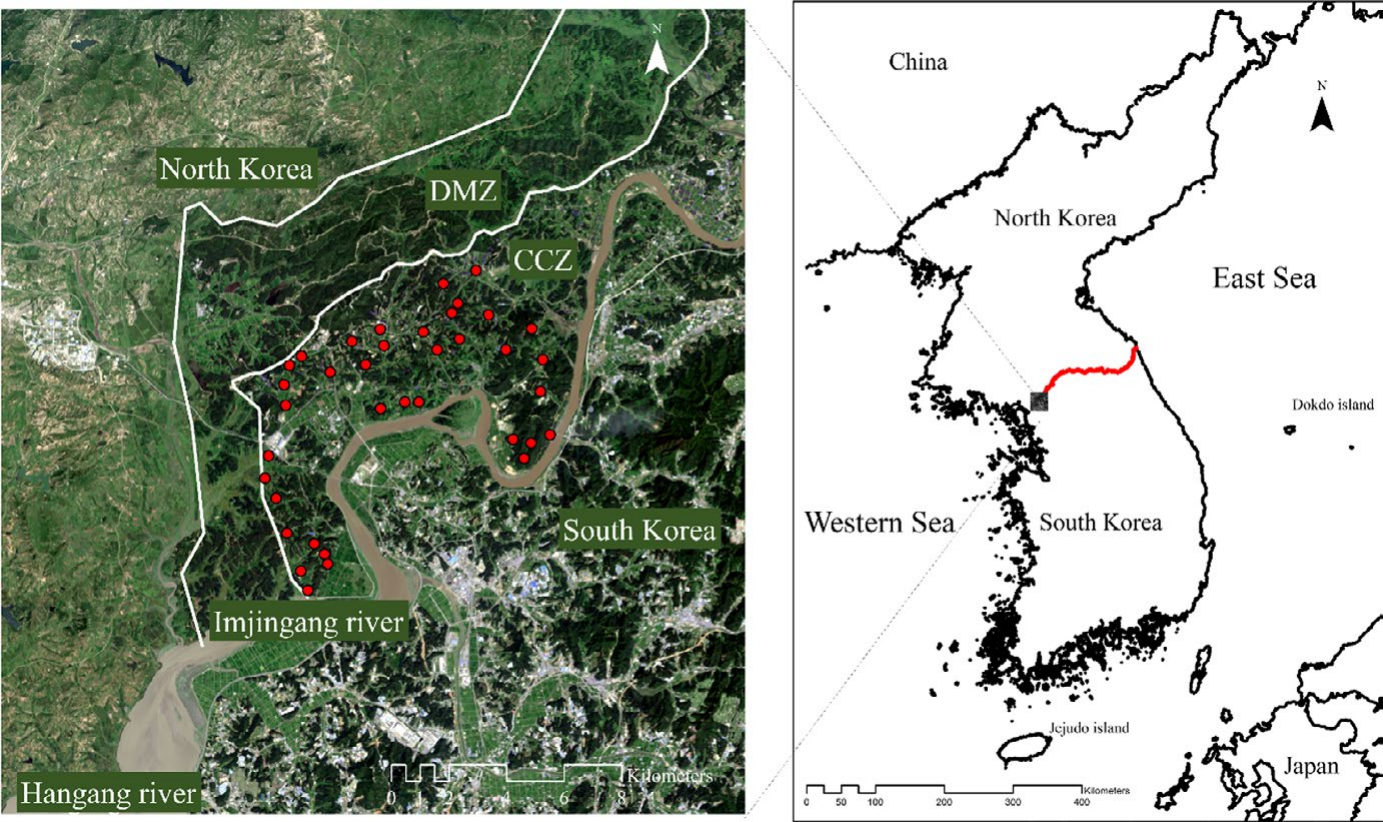
Map showing the study area. Red dots indicate surveyed sites

Summer bird surveys were conducted in the fourth week of July and the first week of August in 2015 and 2016. This period was a break from farming, and thus the impact by anthropogenic factors was expected to be minimal. In addition, the season would be the postbreeding season, and thus the juvenile birds could be involved in the surveys. Study site located in the CCZ, Paju‐si, Gyeonggi‐do Province, South Korea (38°00′N 126°51′E, 37°49′N 126°40′E; Figure 1). Thirty‐six irrigation ponds were randomly chosen among 500 irrigation ponds in the study site to examine the influence on the avian community against drought. The survey was performed in the morning (5:30–7:30 a.m.), midday (11:00 a.m.–1:00 p.m.), and afternoon (5:30–7:30 p.m.) at each site using the line transect method. During the monitoring period, investigators were divided into two teams. Each team surveyed along the 500‐m monitoring line, which began at the survey point (pond). Teams then moved along either side, marking the occurrence and distribution of bird species on a map while conducting the field survey. The average transit time was approximately 30 min per site. Birds were recorded within 100 m‐wide range from the monitoring line.

All the survey sites included an irrigation pond, but the composition of landscape elements around the pond differed undesigningly. The LULC type was calculated based on 1.0 × 1.5 km images along the monitoring line, which has 500‐m radius‐wide buffer, using Google Earth image (RGB band). The RGB image was segmented using eCognition (segmentation: shape 0.3, compact: 0.5) and classified manually. The classification results were verified on the spot. Land use type was classified in nine categories: rice paddy (RICE); ginseng field (GF); common field‐cultivated soybean, adlay, etc. (F); barren field (BF); irrigation pond (POND); freshwater, including river and stream (RIVER); forest (FOREST); artificial structures, including agricultural facilities, army camp, buildings, roads, etc.; AS); and mixed vegetation (MV; including weeds, ruderals on ditches and fallows).

The number of TAL types was determined using the NbClust package (Charrad et al., 2014), with the optimal number being determined according to whether they provided 30 metrics in the dataset for use in the evaluations. Clustering was based on ward distance and the vegan package. Using the prcomp package to summarize the classified LULC characteristics as a single axis, the PCA analysis was performed.

### 2.2 Drought index

The study sites experienced droughts in 2015 and 2016. The average annual precipitation in the region from April to July is 718 mm. However, the precipitation amounts from April to July in this area in 2015 and 2016 were 393 and 654 mm, respectively. When the precipitation is low from April to July, rice paddies usually would be supplied with less water, compromising their ability to function as wetlands. In 2015, precipitation was 40% lower than in 2016 and 45% lower than the average precipitation over the previous 10 years.

The degree of drought was examined using the standardized precipitation evapotranspiration index (SPEI). The SPEI, an index that improved the existing drought index to make apparent effects attributable to climate change, was first proposed by Vicente‐Serrano et al. (2010). Unlike other drought indices, rather than simply calculating the precipitation (P), the SPEI uses climatic water balance, with the difference between precipitation and reference evapotranspiration (P–ET0) being used as the input. This allows us to more precisely identify extreme temperature rises or changes in drought caused by heat waves (Beguería et al., 2014).

With a value for potential evapotranspiration (PET), the difference between the precipitation (P) and PET for the month *i* is calculated: *D_i_* = *P_i_* − PET*_i_* which provides a simple measure of the water surplus or deficit for the analyzed month. The calculated *D_i_* values are aggregated at different time scales.

$$\text{SPEI}=W‐\frac{C_{0}+C_{1}W+C_{2}W^{2}}{1+d_{1}W+d_{2}W^{2}+d_{3}W^{3}},$$

where *W* = −2ln(*p*) for *p* ≤ .5, *p* being the probability of exceeding a determined *D* value, *p* = 1 − *F*(*x*). If *p* > .5, *p* is replaced by 1 – *p* and the sign of the resultant SPEI is reversed. The constants are as follows: *C*_0_ = 2.515517, *C*_1_ = 0.802853, *C*_2_ = 0.010328, *d*_1_ = 1.432788, *d*_2_ = 0.189269, and *d*_3_ = 0.001308. The average value of the SPEI is 0, and the standard deviation is 1. The SPEI is a standardized variable, and it can therefore be compared with other SPEI values over time and space. An SPEI of 0 indicates a value corresponding to 50% of the cumulative probability of *D*, according to a Log‐logistic distribution.

In this study, using the data of the monthly cumulative precipitation, average maximum temperature, and average minimum temperature (Korea Meteorological Administration, 2019), 3‐month time scales were used. The period was calculated for a period of 10 years (2008–2018). Data were analyzed by the SPEI package in R 3.5 (Begueria & Vicente‐Serrano, 2017).

### 2.3 Taxonomic and functional diversity

In this study, species richness (SR) was used as a measure of taxonomic diversity (Magurran, 2013) for each site. The avian diversity was represented by the maximum number of species among the three surveys which were recorded on each site.

Functional diversity was calculated based on several traits (see Cagan, 2006; Flynn et al., 2009; Luck et al., 2012): diet, foraging location, habitat location, nesting location, migration status, and morphological features (body mass), which have been commonly used in previous studies (Table 1). Diet, foraging location, and morphological features are continuous traits (Wilman et al., 2014), and habitat location, nesting location, and migration status are categorical traits (Lee et al., 2000; Takagawa et al., 2011). For analysis, the categorical data were converted to binary values.

**Table 1:** Summary of traits and avian functional diversity indices

Category	Trait	Units and trait type
Diet	Invertebrates, Vertebrates, Herptile, Fish, Unknown, Decaying biomass, Fruit, Nectar, Seed, Plant	Percentage, continuous
Foraging location	Water (below surface, around surface), Ground, Tree (understory, mid‐high, canopy), Aerial	Percentage, continuous
Morphology	Body mass	Grams, continuous
Migrant	Migration (migratory status in the summer)	1: Migratory, 0: Resident, binary
Habitat location	Urban area, Agricultural area, Coast, Lake‐river, Wetland, Grassland, Forest, Mountain	1: Yes, 0: No, binary
Nesting location	Ground, Shrub, Tree, Tree hole, Cliff, Water level, Artificial structure, Deposition	1: Yes, 0: No, binary

Functional richness (FRic), functional evenness (FEve; Villéger et al., 2008), and functional divergence (FDiv; Mouchet et al., 2010) were calculated using the FD package in R (Laliberté et al., 2014). FRic refers to the amount of functional space occupied by species in a community. FEve indicates the distribution of species abundance in a community within a functional space. FDiv describes the difference in distance from the center of the functional space and is related to species abundance measures.

### 2.4 Statistical analysis

Differences across the LULC types, drought event occurrence, and diversity indices were evaluated by Bayesian linear mixed models using MCMCglmm (Hadfield, 2010), running 130,000 iterations with a burn‐in period of 30,000 and a thinning interval of 100. Autocorrelation was <.02. Sites and months were modeled as random effects, and drought and land configuration for each functional diversity and species richness index were included as the main fixed effects.

The effect of drought on the functional diversity and taxonomic diversity for each LULC type was evaluated by paired *t* test and the Wilcoxon signed‐rank test. The relative ratio of the LULC in the TAL types was evaluated by ANOVA or Kruskal–Wallis test and post hoc test Tukey's HSD or Conover post hoc test. The relationship with LULC and diversity indices was tested by Kendall correlation. All statistical analyses were conducted in R 3.5.

## 3 RESULTS

### 3.1 Compositions of TALs and avian diversity

**Figure 2:**
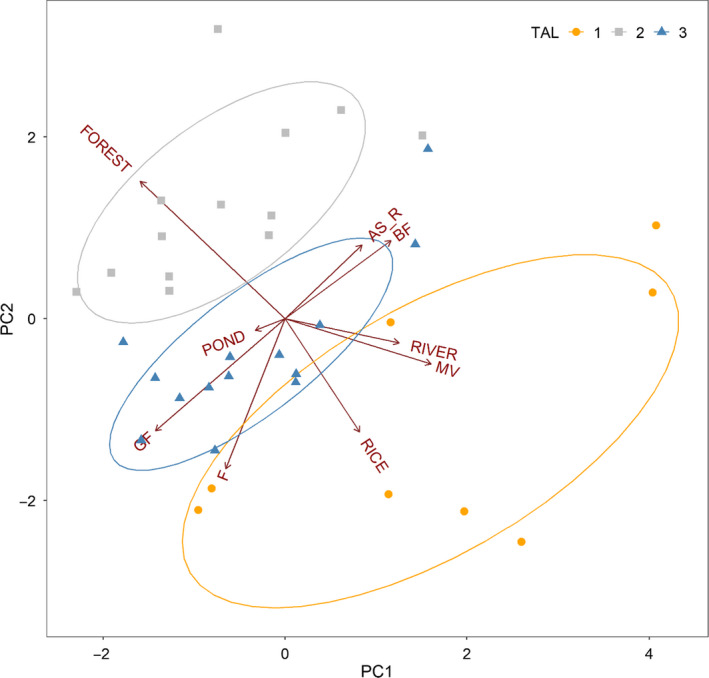
PCA results of the TAL composition. The arrows indicated the direction of the habitat composition. The first two axes explain 48.8% of the variation of the bird assemblages. PC 1 showed a very high correlation with mixed vegetation (MV; *r* = .46), and PC 2 was highly correlated with field (F). The round shape and yellow of the dot is TAL 1, and the gray square point is TAL 2. TAL 3 is the blue triangle point

Based on the clustering, the characteristics of study sites were categorized into three types (Figure 2 and Figure S1). The cumulative proportion of the first axis and the second axis of PCA was 49% (Figure 2, Table 2). The PC 1 axis explained 27% of variance and showed a positive correlation with mixed vegetation (MV; *r* = .46) and a negative correlation with forest (FOREST; *r* = −.46). The PC 2 axis explained 22% of variance and was highly correlated with field (F; *r* = −.57; Table 2). TAL 1 appeared to be characterized by wetland such as rice paddies (RICE) and waterbody (RIVER) which were significantly higher than other TAL (Figure 2 and Figures S1–S3). TAL 2 had high proportion of forest (FOR) which was larger than any other TAL type. TAL 3 was represented by high proportion of dryland like field (F), ginseng field (GF), and barren field (BF).

**Table 2:** Principal Component Analysis results showing TAL variables with correlation in the PC1 and PC2 ordination axis

		PC1	PC2
The correlation with axis and variation	AS_R	0.24	0.26
	BF	0.34	0.27
	F	−0.19	−0.53
	FOREST	−0.46	0.48
	GF	−0.41	−0.39
	MV	0.46	−0.16
	POND	−0.10	−0.04
	RICE	0.24	−0.40
	RIVER	0.36	−0.08
Importance of components	Standard deviation	1.553	1.4058
	Proportion of variance	0.27	0.22
	Cumulative proportion	0.27	0.49

**Figure 3:**
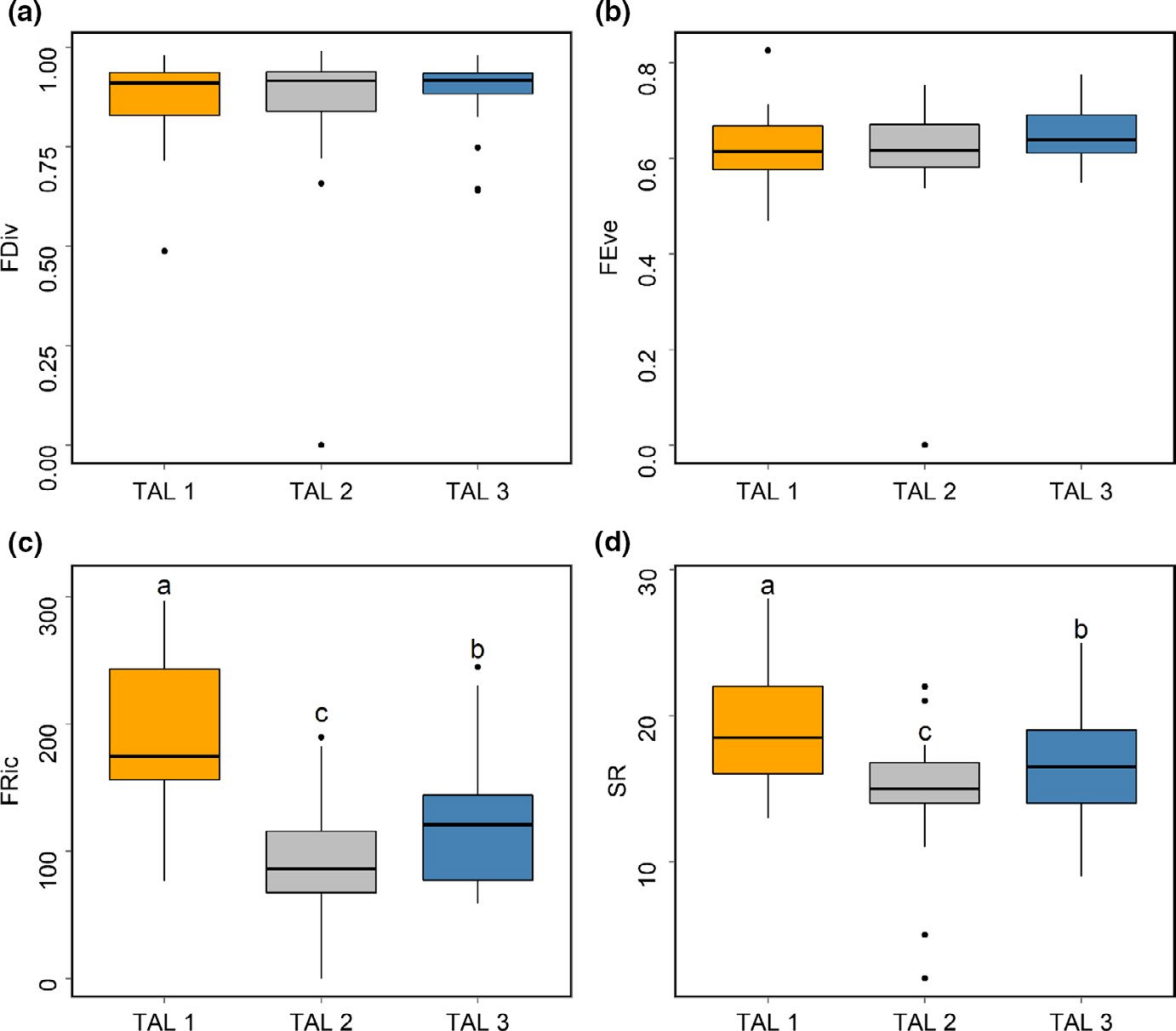
Diversity indices of the TAL types. For each type of TAL, only the FRic and SR differ statistically (*p* < .05, MCMCglmm). (a) The differences in FDiv by TAL types (*p* > .05, MCMCglmm). (b) The differences in FEve by TAL types (*p* > .05, MCMCglmm). (c) The differences in FRic by TAL types (*p* < .05, MCMCglmm). (d) The differences in SR by TAL types (*p* < .05, MCMCglmm). The yellow box plot is TAL1, the gray box plot is TAL2, and the blue box plot is TAL 3

FEve and FDiv did not significantly differ by TAL type. However, SR and FRic varied significantly depending on TAL types. TAL 1 was associated with the highest SR and FRic scores (*p*MCMC < .001, Figure 3). TAL 2 showed the lowest SR and FRic scores (*p*MCMC < .001).

### 3.2 Drought and diversity indices of TAL

**Figure 4:**
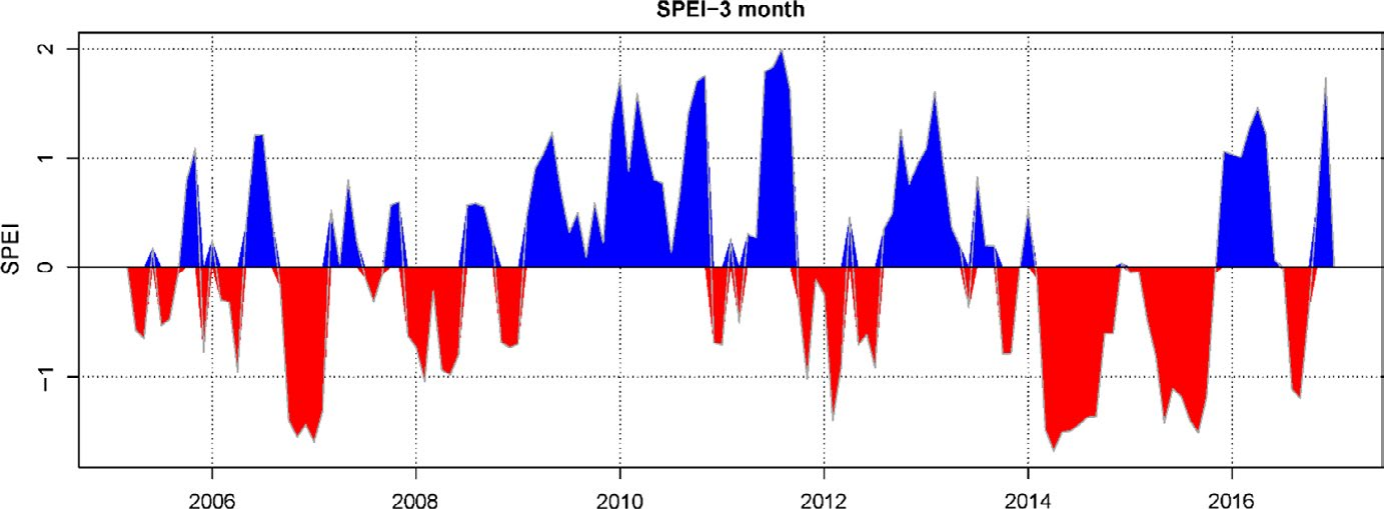
The result of the SPEI (standardized precipitation evapotranspiration index) analysis at 3‐month time scale. In 2014–2015, there was a continuous drought for two years, unlike in the previous 10 years. During the past decade, droughts exceeding 12 month have occurred periodically. Red area indicates droughts

**Figure 5:**
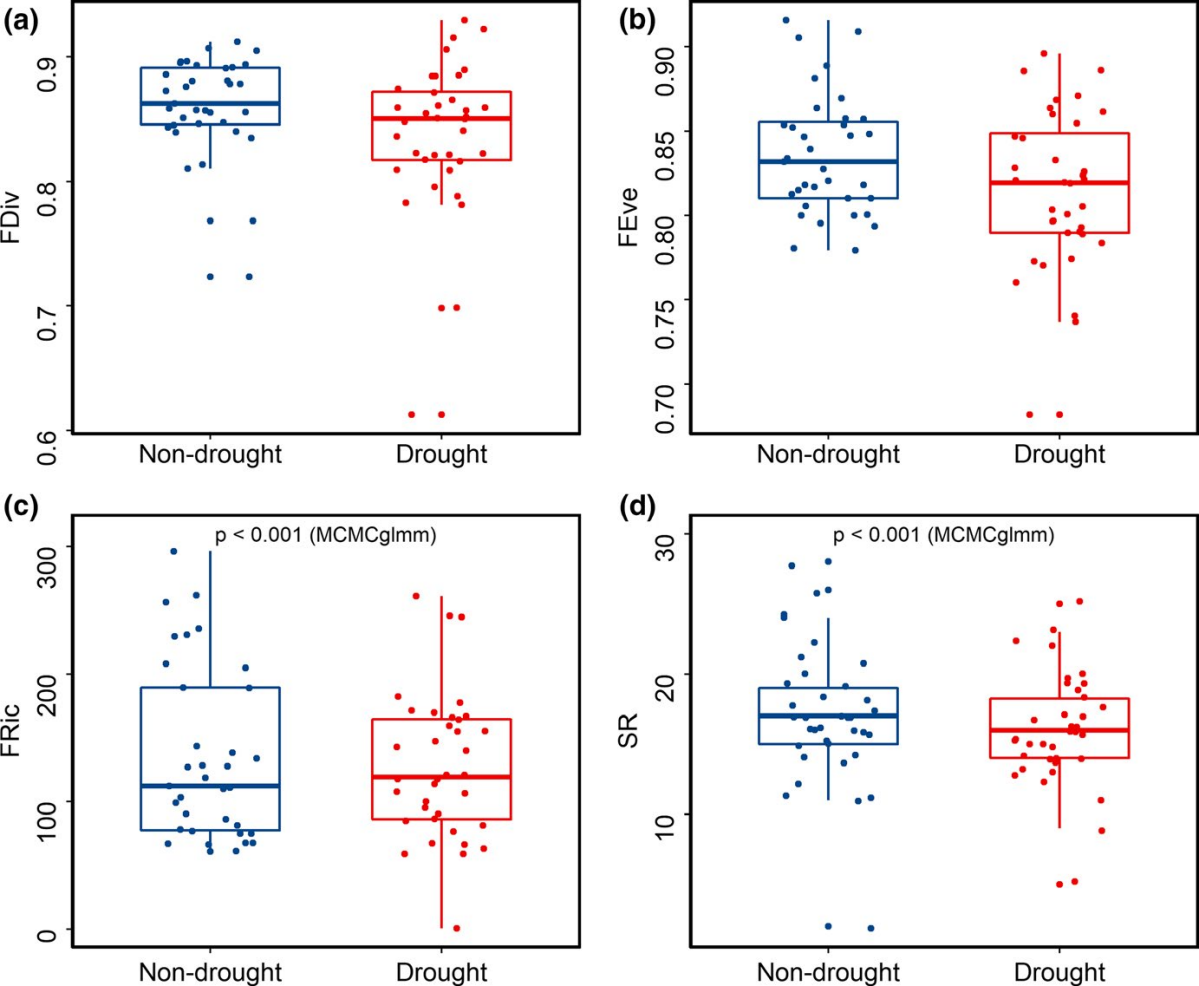
Diversity indices with drought events. For each drought condition of Diversity indices, only the FRic and SR differ statistically (*p* < .05, MCMCglmm). (a) The differences in FDiv by drought condition (*p* > .05, MCMCglmm). (b) The differences in FEve by drought condition (*p* > .05, MCMCglmm). (c) The differences in FRic by drought condition (*p* < .05, MCMCglmm). (d) The differences in SR by drought condition (*p* < .05, MCMCglmm). The blue box plot is nondrought, and the red box plot is drought condition

SPEI values showed that drought occurred periodically over the decade (Figure 4), but severe drought in 2014 and 2015 was followed in 2016 by rainfall within a normal range. Overall, the diversity indices in nondrought year were higher than those in drought year (Figure 5). In addition, there were significant differences between drought and nondrought years on FRic and SR (MCMCglmm, Table S1).

**Figure 6:**
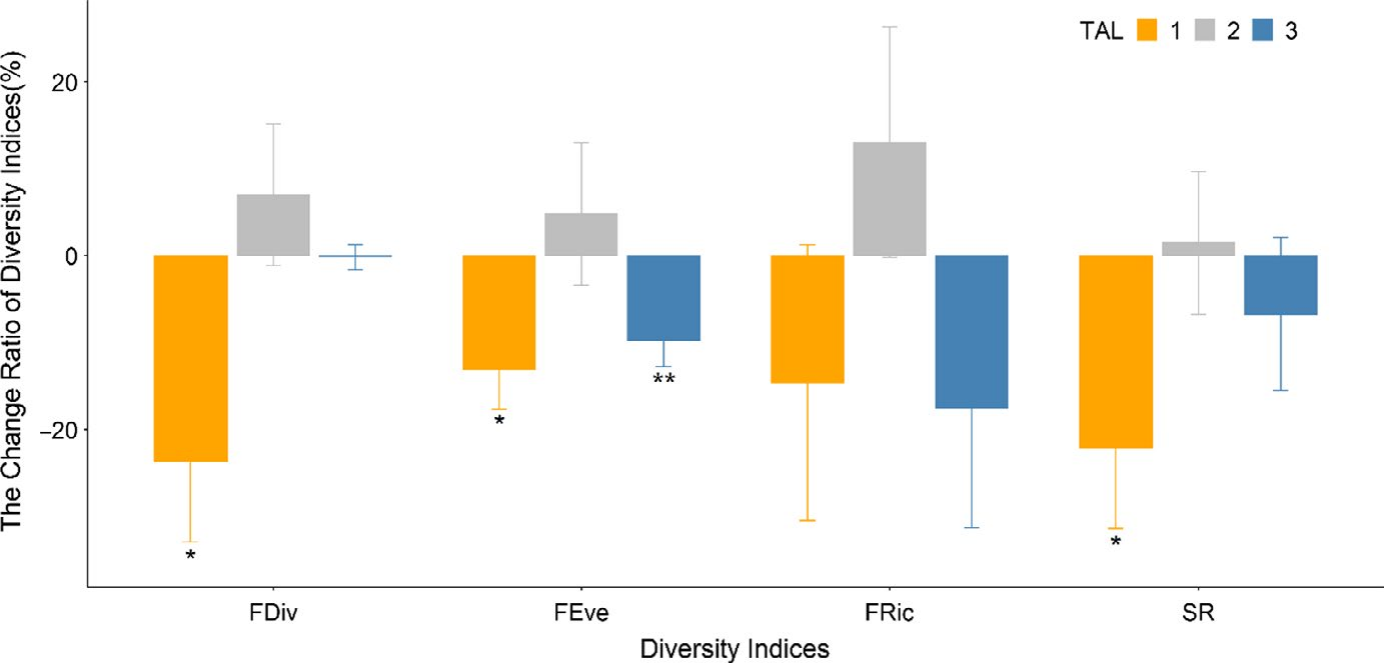
Changes in the biodiversity indices of birds with a drought event. The yellow bar is TAL1, the gray bar is TAL2, and the blue bar is TAL 3. (*p < .05, **p < .01)

The impact of drought on bird populations differed by TAL type (Figure 6). In TAL 1, the diversity indices that were significantly reduced during a drought were FDiv, FEve, and SR. FDiv decreased by 23.6% (Wilcoxon's signed‐rank test, *p* = .01). FEve diminished by 13.1% (paired *t* test, *t*_8_ = 2.84, *p* = .02), and SR decreased by 22.1% (Wilcoxon's signed‐rank test, *p* = .03). TAL 2 did not show a statistically significant difference in diversity indices due to drought. TAL 3 showed a significant 9% reduction in FEve during drought (paired *t* test, *t*_13_ = 3.06, *p* = .009). Furthermore, as to the relationship between the diversity indices, LULC type, and drought event, 5 LULC elements of FOREST, RICE, GF, MV, and RIVER had negative or positive correlation (*p* < .05; Figure S4). When the proportion of FOREST increased, the differences in diversity indices between drought and nondrought decreased. The gap of diversity indices between drought and nondrought events was increased with RICE, MV and RIVER increased. Besides, unlike our prediction, there was no significant difference in the relationship with the proportion of POND and diversity indices (*r* < .22).

## 4 DISCUSSION

This study showed that the bird community responded to the drought events in the TAL. Usually, the landscape with high proportion of rice paddies showed the highest avian SR and functional diversity, but species assemblages lack resilience to drought. In contrast, TAL with forest cover had lower average diversity but provided greater resilience to drought events. As a result, we can suggest that forests play an important role in the Korean TAL with *dumbeong*, and the maintenance of forests in the TAL is key to maintaining avian species diversity.

The previous studies showed that functional diversity and taxonomic diversity of birds appeared differently according to the land use type (Martin et al., 2012; Morelli et al., 2018). Our study had similar results that a significant difference occurred in the diversity of birds according to the TAL type. In particular, rice paddy fields supported the highest richness, as found elsewhere (Elphick, 2000; Tourenq et al., 2001) Meanwhile, the FEve and FDiv showed no significant difference depending TAL type in our study. It was also found in previous studies (Lee & Martin, 2017; Morelli et al., 2018). This is due to the measurement mechanisms of FEve and FDiv. The uniformity of species in functional space is scribed by FEve—the more regularly distributed the functional distance among species, the lower the FEve score (Mason et al., 2005; Villéger et al., 2008). In other words, the range of niche space of community would be limited to the utilization of the entire range of resources available. FDiv was calculated the extent to which the distribution of quantities in niche space maximizes the divergence of functional properties within the community (Mason et al., 2005; Villéger et al., 2008). The low FDiv indicates that the degree of niche differentiation was simplified, thus leading to high competition. Our results suggested that, regardless of TAL types, the feed resource which was exploted in avian assemblage could be much the same (FEve), and the degree of ecological niche differentiation in the avian assemblage was almost the same (FDiv). On the other hand, in general, functional richness increases as the species abundance increases. Our research results also showed the similar trend of high correlation. It is because the formula to analyze functional richness is not independent from species richness (Mason et al., 2005).

As high as richness is, TAL1, which is not seen as having high structural stability, can be seen as instantly responding communities to droughts. TAL 1 biodiversity diminished after drought. Even though rice paddies function as a semi‐natural wetlands, paddies and *dumbeongs* differ in this respect from natural wetlands. In previous study, functional diversity raised in drought conditions, presumably because lower water levels expose valuable food resources for birds in natural wetlands, such as floodplains (Almeida et al., 2017). Organisms utilizing rice paddies can be more likely to be vulnerable to drought occurrence than those using natural wetlands. Thus, during a drought, resource availability becomes more depleted in rice paddies than in natural wetlands.

Meanwhile, surprisingly, there was no correlation between the relative proportion of *dumbeong* and the resilience of each TAL type. We expected *dumbeong* to act functionally like natural wetlands at drought events but in this study we were unable to demonstrate this. Presumably, it seems that the water capacity of each *dumbeong* was insufficient to address the very severe drought and that the *dumbeong* did not serve as a refuge for the wildlife. We could easily find dry rice fields and *dumbeongs* during the 2015 survey. Droughts have been relatively common in Korea in recent years, but drought in 2015 occurred consecutively in 2014 and was particularly severe. During the relatively dry period, *dumbeong* helped to maintain the paddy ecosystem and protected the wildlife in itself, but it seems that *dumbeong* could not play a role in the severe drought. Instead, forest may effectively contribute to maintaining avian diversity against severe drought rather than the *dumbeong* size.

The forest, rather than *dumbeong*, appears to affect the stability of the bird communities. The SR and FRic for TAL2 were lower than for the other types, but in the case of drought, the diversity of bird communities showed a positive, albeit not statistically significant change. Consistent with this, droughts positively affected bird diversity in the forest areas in a recent study (Albright et al., 2010). Bird populations exploiting forest may experience a time lag in breeding than those utilizing paddy fields, as the food resources including vegetation structure does not change immediately to cope with a drought or high temperature (Bertrand et al., 2011; Kissling et al., 2010). Thus, how bird communities respond to drought varied, at least temporarily, depending on habitat properties. Interestingly, there was similar structure based on trait between TAL type (Figure S5).

As droughts are more likely to occur due to climate change, increasing the stability of bird communities in traditional agricultural landscapes in East Asia should be a priority. Rice paddies, which make up a large part of the traditional landscape of East Asia, provide water and a variety of resources of food to birds, maintaining high biodiversity in the absence of drought conditions. However, our study suggested that maintaining forests patches in the agricultural area may retain ecological niches when drought occurs. Previous studies highlighted that maintaining and restoring forest areas should be a key component of avian conservation programs at drought events (Mac Nally et al., 2009). For implications, in order to maintain biodiversity in the western DMZ TAL, it is necessary to restrict the destruction of forests, and where forests have disappeared, restoration to a certain level is needed. The western DMZ region of South Korea is threatened by increased cultivation of ginseng, an important commodity crop (Park & Nam, 2013). For this reason, the existing forests may be converted into farmland (Kim, unpublished data). Furthermore, improved North‐South Korea relations offer the possibility of development of the DMZ but such plans need to encompass safeguards for the conservation of biodiversity. We hope that our study may provide one basis for such plans.

## CONFLICT OF INTEREST

None declared.

## AUTHOR CONTRIBUTION

**Jae Hyun Kim:** Conceptualization (lead); Data curation (lead); Formal analysis (lead); Funding acquisition (supporting); Investigation (equal); Methodology (lead); Project administration (supporting); Visualization (lead); Writing‐original draft (equal); Writing‐review & editing (equal). **Shinyeong Park:** Investigation (supporting); Project administration (supporting); Software (supporting), Writing‐original draft (equal); Writing‐review & editing (equal). **Seung Ho Kim:** Funding acquisition (equal); Investigation (equal); Project administration (equal); Resources (equal); Validation (equal). **Keunwon Kang:** Investigation (equal). **Bruce Waldman:** Validation (supporting); Writing‐review & editing (supporting). **Myung Hwa Lee:** Funding acquisition (supporting); Investigation (supporting); Resources (supporting). **Minhye Yu:** Investigation (supporting); Software (supporting). **Hyunyoung Yang:** Investigation (supporting). **Hyun Yong Chung:** Investigation (supporting). **Eun Ju Lee:** Funding acquisition (equal); Project administration (equal); Resources (equal); Supervision (lead); Validation (equal).

## Supporting information

Supplementary Material

## Data Availability

https://doi.org/10.5061/dryad.02v6wwq1n
