# Supplementary material for: Structural implications of traditional agricultural landscapes on the functional diversity of birds near the Korean Demilitarized Zone
Source: Ecol Evol. 2020 Oct 12;10(23):12973–82. doi: 10.1002/ece3.6880 (PMC7713986; doi:10.1002/ece3.6880)
Supplement: Supplementary file 1 — Supplementary Material [file ECE3-10-12973-s001.docx]

Structural implications of traditional agricultural landscapes on the functional diversity of birds near the Korean Demilitarized Zone

Jae Hyun Kim^1,2^, Shinyeong Park^1,2^, Seung Ho Kim^2^, Keunwon Kang^2^, Bruce Waldman^1,3^, Myung Hwa Lee^2^, Minhye Yu^2^, Hyunyoung Yang^1^, Hyun Yong Chung^2^, Eun Ju Lee^1*^

^1^ School of Biological Sciences, Seoul National University, Seoul 08826, Republic of Korea

^2^ DMZ Ecology Research Institute, Paju 10881, Republic of Korea

^3^ Department of Integrative Biology, Oklahoma State University, Stillwater, Oklahoma 74078, United States of America

*Corresponding author: ejlee@snu.ac.kr (+82-10-6525-1962)

**Appendices**

**Table S1 MCMC glmm including bird functional and taxonomic diversity as the dependent variables and TAL and drought condition as the independent variables. Abbreviations are as follows: post.mean = posterior mean, l-95 % CI = lower limit of the 95 % credible interval, u-95 % CI = upper limit of the 95 % credible interval, eff.samp = effective size of the sample, and *p*MCMC = posterior probability for the parameter not being different from zero. The prior was defined as follows: list(R = list(V = 1, nu = 0.002), G = list(G1 = list(V = 1, nu = 0.002), and G2 = list(V = 1, nu = 0.002))).**

| Factor | | post.mean | l-95 % CI | u-95 % CI | eff.samp | *p*MCMC |
| --- | --- | --- | --- | --- | --- | --- |
| FRic | TAL2 | -124.04 | -126.17 | -124.09 | 1000 | <0.001 *** |
|  | TAL3 | -85.85 | -87.15 | -84.61 | 1000 | <0.001 *** |
|  | Drought | -3.43 | -3.88 | -2.98 | 1000 | <0.001 *** |
| FEve | TAL2 | -0.017 | -0.56 | 0.63 | 1000 | 0.918 |
|  | TAL3 | 0.019 | -0.55 | 0.61 | 1000 | 0.962 |
|  | Drought | 0.002 | -0.47 | 0.45 | 1000 | 0.986 |
| FDiv | TAL2 | -0.025 | -0.67 | 0.55 | 1000 | 0.928 |
|  | TAL3 | 0.005 | -0.59 | 0.60 | 1000 | 0.980 |
|  | Drought | -0.002 | -0.42 | 0.47 | 1118 | 0.982 |
| SR | TAL2 | -4.91 | -5.89 | -3.91 | 1000 | <0.001 *** |
|  | TAL3 | -3.13 | -4.33 | -2.05 | 1000 | <0.001 *** |
|  | Drought | -0.92 | -1.37 | -0.47 | 1000 | <0.001 *** |

**Table S2 Species list and average population of avian species size by TAL types.**

| Order | family | Scientific name | Common Name | TAL1 | TAL2 | TAL3 |
| --- | --- | --- | --- | --- | --- | --- |
| Anseriformes | Anatidae | *Aix galericulata* | Mandarin Duck | 2.0 | 4.0 | 2.0 |
|  |  | *Anas poecilorhyncha zonorhyncha* | Chinese Spot-billed Duck | 11.6 | 2.3 | 2.4 |
| Charadriiformes | Scolopacidae | *Actitis hypoleucos* | Common Sandpiper | 1.0 |  |  |
|  |  | *Tringa ochropus* | Green Sandpiper | 1.0 | 1.0 |  |
| Ciconiiformes | Ardeidae | *Ardea cinerea jouyi* | Grey Heron | 1.7 |  |  |
|  |  | *Ardeola bacchus* | Chinese Pond Heron |  |  | 1.0 |
|  |  | *Bubulcus ibis coromandus* | Cattle Egret | 2.8 | 3.8 | 4.2 |
|  |  | *Butorides striata amurensis* | Striated Heron | 1.7 | 1.0 | 1.0 |
|  |  | *Egretta alba modesta* | Great Egret | 10.5 | 6.0 | 4.6 |
|  |  | *Egretta garzetta garzetta* | Little Egret | 1.7 | 2.5 | 1.2 |
|  |  | *Nycticorax nycticorax nycticorax* | Black-crowned Night Heron | 1.7 | 1.0 | 2.3 |
| Columbiformes | Columbidae | *Streptopelia orientalis orientalis* | Oriental Turtle Dove | 8.1 | 6.7 | 5.6 |
| Coraciiformes | Alcedinidae | *Alcedo atthis bengalensis* | Common Kingfisher | 1.4 | 1.0 | 1.2 |
|  |  | *Halcyon coromanda major* | Ruddy Kingfisher | 2.3 | 1.3 | 1.0 |
|  |  | *Halcyon pileata* | Black-capped Kingfisher | 1.6 | 1.8 | 1.6 |
|  | Coraciidae | *Eurystomus orientalis* | Oriental Dollarbird | 6.7 | 4.1 | 6.1 |
| Cuculiformes | Cuculidae | *Cuculus canorus canorus* | Common Cuckoo | 1.3 | 2.4 | 2.4 |
|  |  | *Cuculus saturatus horsfieldi* | Oriental Cuckoo |  |  | 1.0 |
| Falconiformes | Accipitridae | *Accipiter nisus nisosimilis* | Northern Sparrow Hawk |  | 1.0 | 2.0 |
|  |  | *Accipiter soloensis* | Grey Frog Hawk | 1.8 | 2.3 | 2.2 |
|  |  | *Butastur indicus* | Grey-faced Buzzard Eagle |  | 1.0 | 1.0 |
|  | Falconidae | *Falco subbuteo subbuteo* | European Hobby | 1.0 | 1.0 |  |
|  |  | *Falco tinnunculus interstinctus* | Common Kestrel | 1.5 | 1.0 | 1.3 |
| Galliformes | Phasianidae | *Coturnix japonica* | Japanese Quail | 4.0 | 2.0 |  |
|  |  | *Phasianus colchicus karpowi* | Ring-necked Pheasant | 2.7 | 1.9 | 2.2 |
| Gruiformes | Rallidae | *Gallicrex cinerea* | Water Cock | 1.0 | 1.0 | 1.5 |
|  |  | *Porzana fusca erythrothorax* | Ruddy-breasted Crake | 1.0 |  | 1.0 |
| Passeriformes | Aegithalidae | *Aegithalos caudatus magnus* | Rong-tailed Tit | 1.0 | 25.0 | 40.0 |
|  | Corvidae | *Corvus corone orientalis* | Carrion Crow | 3.3 | 5.1 | 2.4 |
|  |  | *Corvus macrorhynchos macrorhynchos* | Large-billed Crow | 1.0 | 4.8 | 2.0 |
|  |  | *Cyanopica cyana koreensis* | Azure-winged Magpie | 2.8 | 4.7 | 1.0 |
|  |  | *Garrulus glandarius brandtii* | Eurasian Jay | 1.5 | 1.4 | 2.0 |
|  |  | *Pica pica sericea* | Black-billed Magpie | 11.3 | 20.0 | 10.2 |
|  | Emberizidae | *Emberiza elegans elegans* | Yellow-throated Bunting | 5.0 | 1.0 | 2.0 |
|  | Fringillidae | *Carduelis sinica ussuriensis* | Grey-capped Greenfinch |  | 1.0 |  |
|  | Hirundinidae | *Hirundo rustica gutturalis* | Barn Swallow | 510.9 | 96.3 | 113.5 |
|  | Laniidae | *Lanius bucephalus bucephalus* | Bull-headed Shrike | 3.1 | 1.3 | 1.4 |
|  |  | *Lanius cristatus lucionensis* | Brown Shrike | 2.0 |  |  |
|  |  | *Lanius tigrinus* | Tiger Shrike | 2.0 | 2.0 |  |
|  | Motacillidae | *Motacilla alba leucopsis* | White Wagtail | 1.6 | 1.5 |  |
|  |  | *Motacilla cinerea robusta* | Grey Wagtail |  | 8.0 | 1.0 |
|  | Muscicapidae | *Ficedula zanthopygia* | Yellow-rumped Flycatcher | 2.0 | 1.0 | 1.0 |
|  |  | *Phoenicurus auroreus auroreus* | Daurian Redstart | 2.1 | 1.7 | 1.7 |
|  |  | *Saxicola torquatus stejnegeri* | Eurasian Stone Chat | 6.0 | 1.0 | 1.6 |
|  | Oriolidae | *Oriolus chinensis diffusus* | Black-naped Oriole | 6.5 | 7.1 | 5.9 |
|  | Paridae | *Parus major mimor* | Great Tit | 38.8 | 34.8 | 30.6 |
|  |  | *Parus palustris hellmayri* | Marsh Tit | 4.6 | 5.1 | 14.2 |
|  | Passeridae | *Passer montanus saturatus* | Eurasian Tree Sparrow | 26.5 | 53.0 | 11.1 |
|  | Pycnonotidae | *Hypsipetes amaurotis* | Brown-eared Bulbul | 10.4 | 7.7 | 7.2 |
|  | Sturnidae | *Sturnus cineraceus* | White-cheeked Starling |  | 1.0 |  |
|  | Sylviidae | *Acrocephalus orientalis* | Oriental Reed Warbler | 1.3 | 1.0 | 7.0 |
|  |  | *Cettia diphone borealis* | Korean Bush Warbler |  |  | 2.0 |
|  |  | *Phylloscopus coronatus* | Eastern Crowned Willow Warbler | 4.7 | 1.0 | 3.0 |
|  | Timaliidae | *Paradoxornis webbianus fulvicauda* | Vinous-throated Parrotbill | 78.0 | 78.2 | 47.0 |
|  | Turdidae | *Turdus hortulorum* | Grey-backed Thrush | 1.0 | 1.5 | 2.0 |
| Pelecaniformes | Phalacrocoracidae | *Phalacrocorax carbo sinensis* | Great Cormorant | 2.0 |  |  |
| Piciformes | Picidae | *Dendrocopos kizuki seebohmi* | Japanese Pygmy Woodpecker | 1.3 | 1.4 | 1.3 |
|  |  | *Dendrocopos leucotos leucotos* | White-backed Woodpecker | 1.7 | 1.7 | 2.0 |
|  |  | *Dendrocopos major japonicus* | Great Spotted Woodpecker | 1.5 | 2.6 | 1.9 |
|  |  | *Picus canus jessoensis* | Grey-faced Woodpecker | 1.6 | 1.6 | 1.9 |
| Strigiformes | Strigidae | *Ninox scutulata japonica* | Brown Hawk-Owl |  | 1.0 | 1.5 |
|  |  | *Otus sunia stictonotus* | Oriental Scops Owl |  | 1.0 |  |


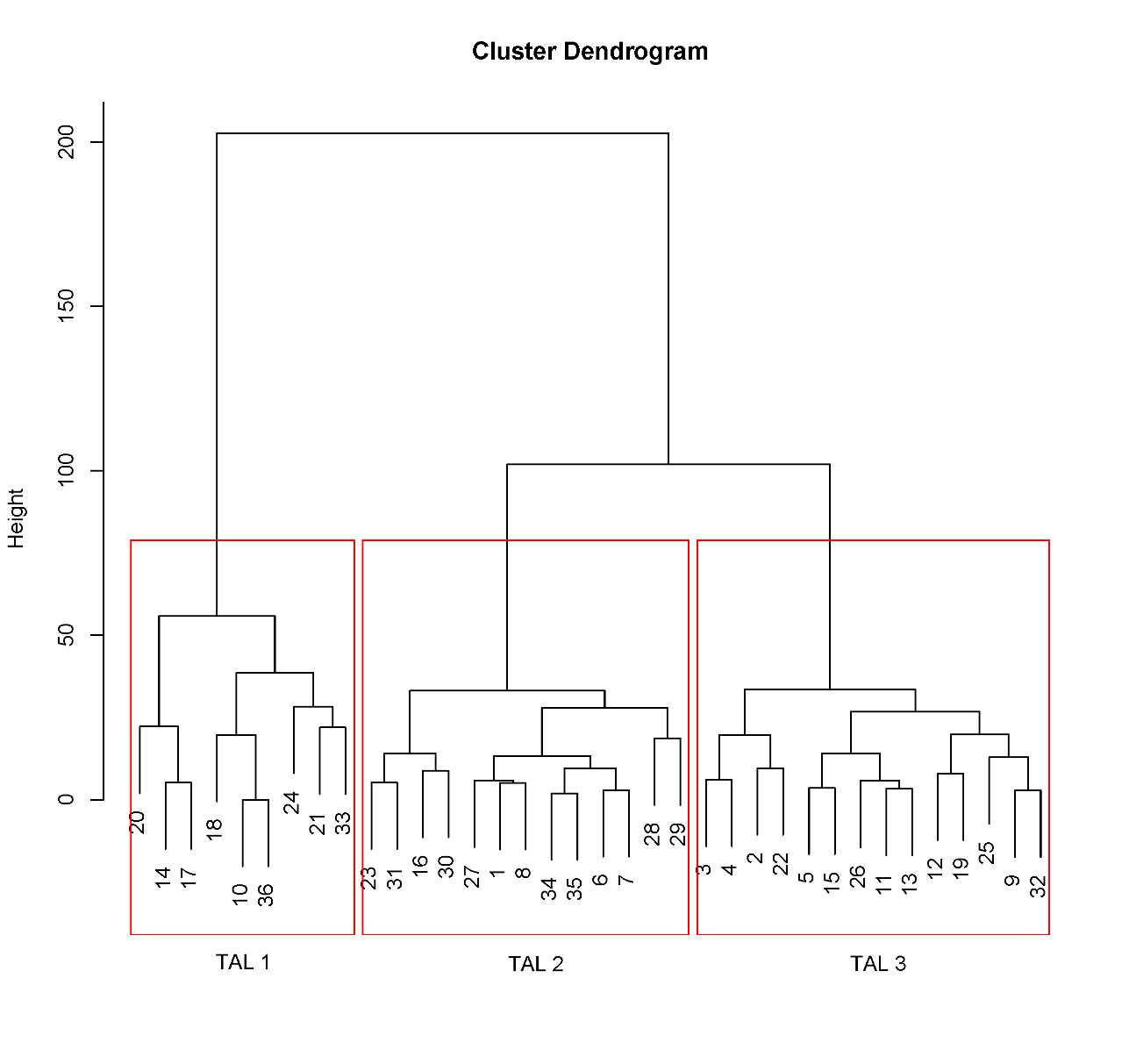


Figure S1 Result of the cluster analysis using ward distance.

**
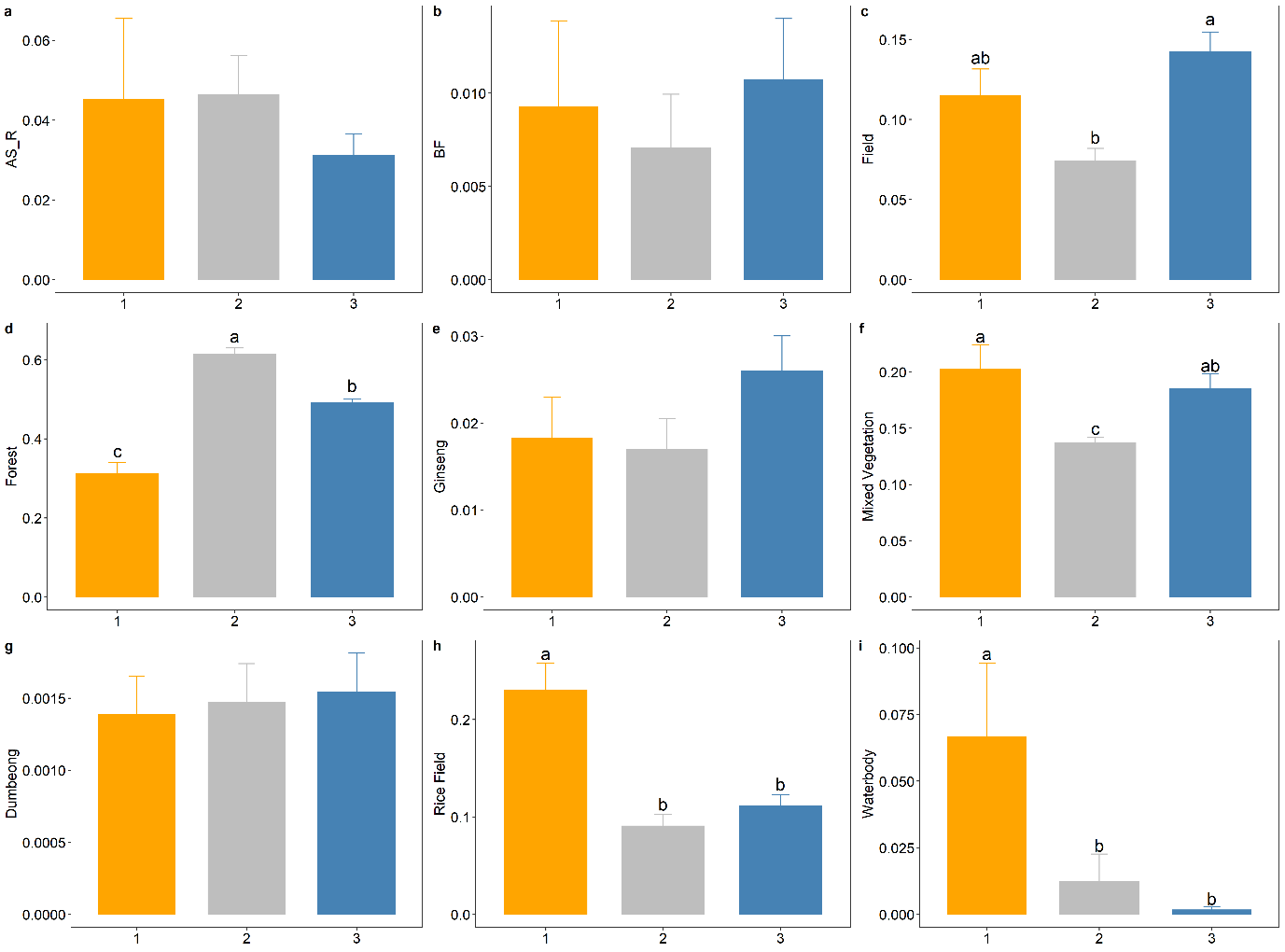
**

Figure S2 Relative ratio of the landscape units in the TAL type. Orange is the TAL 1, Grey is the TAL 2, blue is the TAL2. (Tukey HSD post hoc test or Conover post hoc test, *p* < 0.05). AS_R is artificial structure and road, BF is barren field.


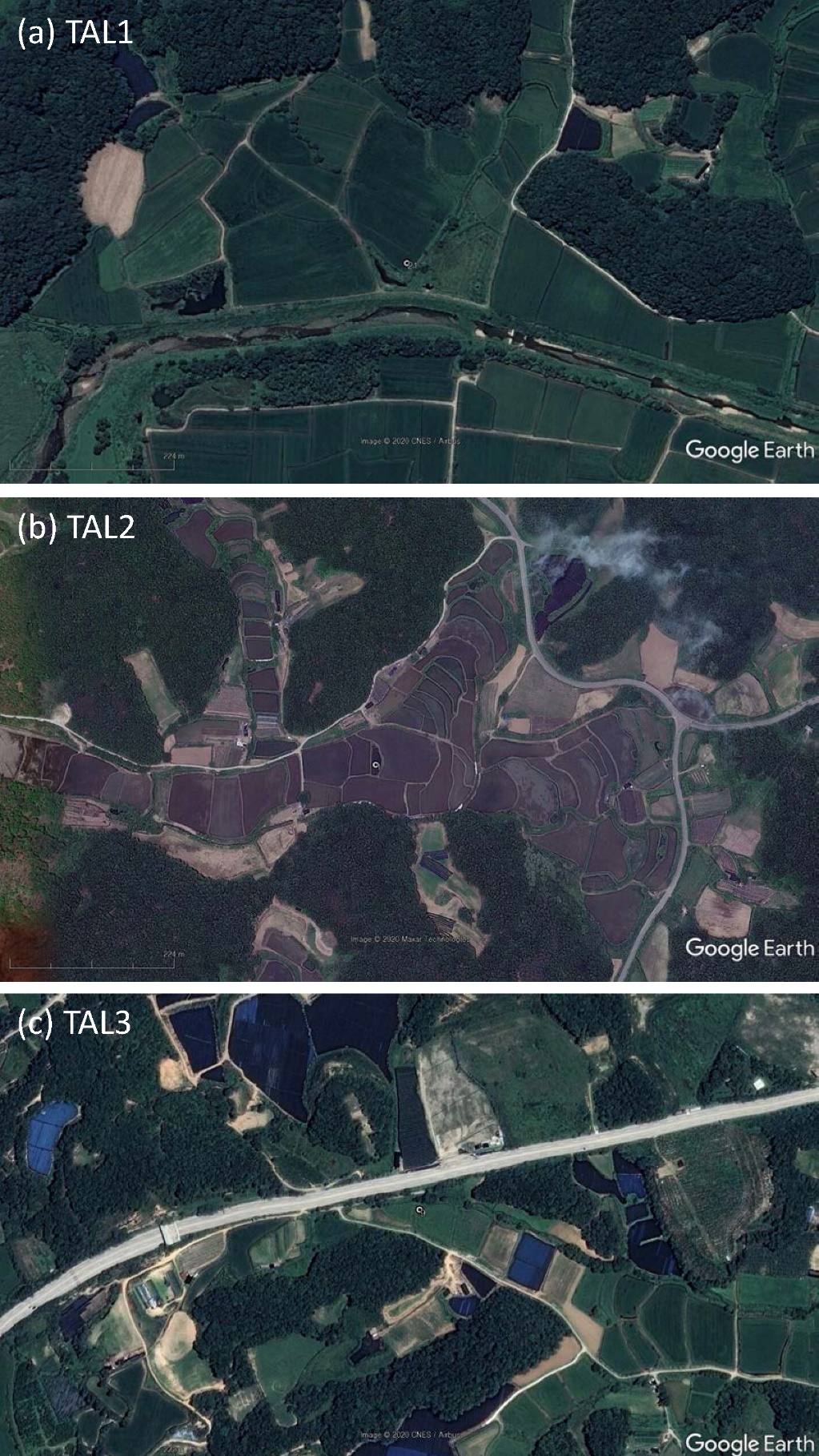


**Figure S3 The example image of TAL types (google earth image).**


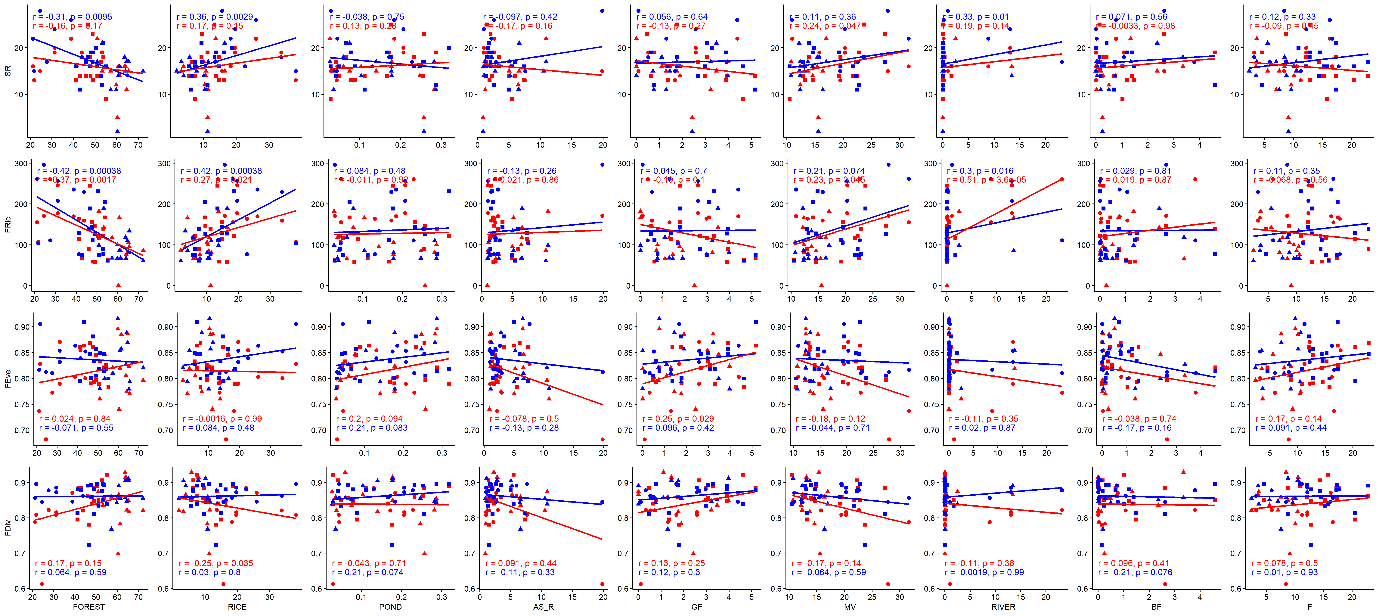


Figure S4 The relationship with LULC (%) and Diverstiy indices. The Red point and line showed the drought condition. The blud point and line showed the non-drought condition. The circle point is TAL1, The triangle point is TAL2, the square point is TAL3. We using Kandall test for correlation.


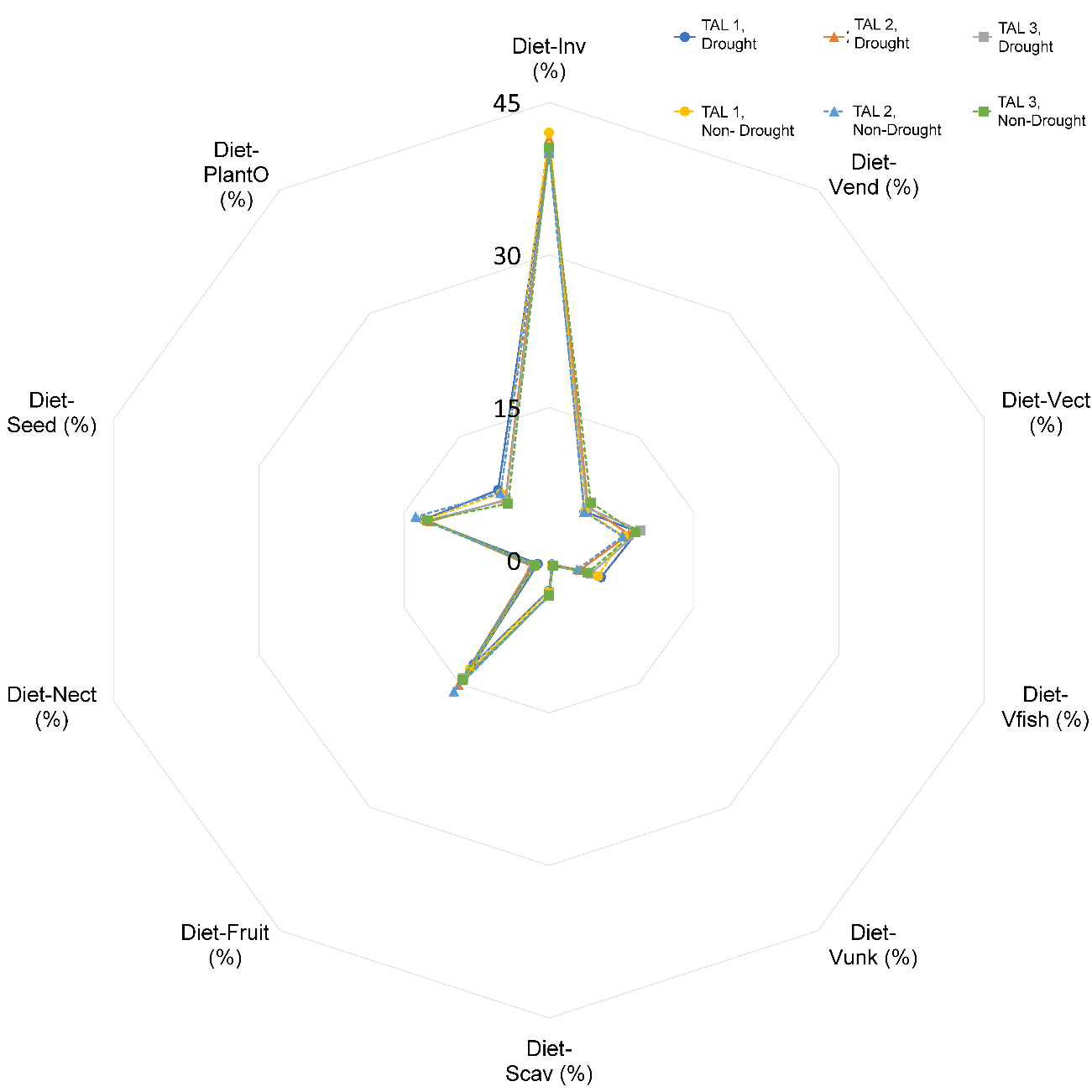


Figure S5 Bird species composition according to the diet types. Dark blue solid line with circle point is TAL 1 under drought event, orange solid line with triangle point is TAL 2 under drought event, Gray solid line with square point is TAL 3 under drought event, yellow dotted line with circle point is TAL 1 under non-drought event, light blue dotted line with triangle point is TAL 2 under non-drought event, green dotted line with square point is TAL 3 under non-drought event. (Diet ~ years*TAL type, ANOVA test, p>0.05)
